# Impact of Nintedanib on tumor angiogenesis and vascular normalization in a mouse model of colorectal cancer

**DOI:** 10.1007/s12672-025-03071-4

**Published:** 2025-07-10

**Authors:** Naita M. Wirsik, Jens M. Seeger, Thomas Schmidt, Frank Hilberg, Hans F. Fuchs, Christiane J. Bruns, Oliver Coutelle, Hamid Kashkar, Lars M. Schiffmann

**Affiliations:** 1 Department of General, Visceral, Thoracic and Transplant Surgery, University of Cologne, Kerpener Straße 62, 50937 Cologne, Germany; 2 Institute for Molecular Immunology, Faculty of Medicine and University Hospital of Cologne, University of Cologne, Cologne, Germany; 3 Boehringer Ingelheim RCV, Doktor-Boehringer-Gasse 5-11, 1120 Vienna, Austria; 4 Center for Integrated Oncology (CIO) Cologne Bonn, Kerpener Str. 62, 50924 Cologne, Germany

## Abstract

**Background:**

For many solid types of cancer including colorectal cancer treatment with the VEGF antibody bevacizumab as anti-angiogenic treatment has become standard of care. Nevertheless, long-term treatment with anti-angiogenic drugs in combination with other treatment modalities or alone can induce resistance through alternative pro-angiogenic pathways. In this study, we investigated the effects of nintedanib, an anti- VEGFR/PDGFR/FGFR kinase inhibitor, on tumor vasculature to determine the potential benefits of combined inhibition of multiple pro-angiogenic factors.

**Methods:**

In a colorectal xenograft model, subcutaneous tumors were treated with Nintedanib. Tumor growth patterns were measured and tumors were analysed histologically regarding effects on tumor angiogenesis and parameters of vascular normalization.

**Results:**

Inhibition of VEGFR/PDGFR/FGFR by Nintedanib was able to reduce tumor growth by significantly inhibiting angiogenesis and inducing tumor cell death. The remaining vessels showed decreased vascular leakage and improved oxygen delivery, indicating a functionally and structurally improved vascular bed resulting from vascular normalization.

**Conclusion:**

In xenograft mouse model of colorectal cancer Nintedanib revealed anti-tumoral effects and induced vascular normalization. Our findings indicate that the treatment with Nintedanib could be able to improve intratumoral oxygen and thereby drug delivery to potentially enhance the efficacy of preexisting oncological therapies such as chemotherapy and radiation.

**Supplementary Information:**

The online version contains supplementary material available at 10.1007/s12672-025-03071-4.

## Introduction

Colorectal cancer (CRC) is the third most common cancer worldwide, with around 1.9 million new cases each year [1]. The 5-year survival rate for CRC is 65%, but this decreases to 14% for patients with metastatic disease [2, 3]. Conventional multimodal treatments for CRC include surgery, radio- and/or chemotherapy, however, these treatments are often not effective in patients with metastatic disease [4].

Optimal anti-angiogenic treatment can augment tumor therapy by normalizing structurally and functionally insufficient tumor blood vessels resulting in improved oxygenation, immune cell infiltration and drug delivery into the tumor [5]. One of the most commonly used anti-angiogenic agents is bevacizumab, a monoclonal antibody that targets VEGF. Bevacizumab has been shown to improve survival in patients with metastatic CRC when used in combination with chemotherapy [6]. However, long-term treatment with bevacizumab can lead to resistance through the upregulation of proangiogenic factors such as fibroblast growth factor (FGF) and platelet-derived growth factor (PDGF) [7–9], necessitating the development of more effective anti-angiogenic agents.

Nintedanib is an oral anti-angiogenic multikinase inhibitor that targets angiogenic escape mechanisms with a very broad inhibitory spectrum that includes proangiogenic VEGF-R 1–3, PDGF-R-α and-β and FGF-R 1–3, and also RET, FLT3, Lck and Lyn6,7 kinases [8, 10, 11]. After a phase III study showed improved progression free survival (PFS) and overall survival (OS) in patients with non-small cell adenocarcinoma of the lung (NSCLC) the combination of Nintedanib with chemotherapy was established as a second- line treatment [12]. More recently, Nintedanib also showed promising results in a placebo-controlled study with modified FOLFOX6 as a second line treatment in metastatic colorectal cancer (mCRC) patients [13].

In this study, we specifically investigated the effects of Nintedanib on tumor angiogenesis and vascular normalization employing a murine xenograft model of colorectal cancer.

## Methods

LS174T human colorectal cancer cells were obtained from Amercian Type Culture Collection (ATCC) and initially gained from adenocarcinoma of the colon. The line is positive for expression of c-myc, N-myc, H-ras, N-ras, Myb, and fos oncogenes, while K-ras was not detected [14].

LS174T human colorectal cancer cells were cultivated as described previously to be further used in animal mice models [15]. In the xenograft model, subcutaneous tumours were generated by injection of LS174T cells (3 × 10^6^ cells) into the flank region of 6–8-week-old female Balb/ cA nude mice (Charles River, Sulzfeld, Germany) subcutaneously. The growth of the tumors was measured every other day. After development of palpable tumour mass, the mice were randomized for the study in groups of mice per treatment arm. Per oral gavage the mice received the anti-human VEGFR/PDGFR/FGFR inhibitor BI-1120 (Nintedanib, Boehringer Ingelheim) at 10 mg/kg (N10, 15 mg/kg (N50) or 100 mg/kg (N100) dissolved in water. The volume of the tumors was calculated using the formular of length 3.14/6 × length × width^2^.

*The* housing of the mice was done by the animal care facility of the University of Cologne under standard pathogen-free conditions including a 12 h light/dark schedule and provisions of food as well as water without any restriction.

### Immunohistochemistry

Tissue samples of the tumors were prepared for microscopical analyses as snap-frozen samples as previously described [16]. For the staining of endothelial cells, cl. caspase 3 and type IV collagen a rat monoclonal anti-mouse CD31 antibody (PECAM-1; 1: 50; clone MEC 13.3, BD Pharmingen, Heidelberg, Germany), a rabbit anti-cleaved caspase-3 (Asp175) antibody (1:200, Cell Signaling, Leiden, The Netherland) and a rabbit polyclonal anti-type IV collagen antibody (1: 100; Clone ab6586, Abcam, Cambridge, UK) were applied as primary antibodies. Pericytes were stained with rat anti-mouse CD140b (PDFGRb) antibody (1:200, eBioscience) and a rabbit anti-NG2 Chondroitin Sulphate Proteoglycan antibody (1:100, Millipore). The detection of CD31 antibody was done by 594-conjugated Alexa Fluor or polyclonalgoat goat anti-rat 647- conjugated antibody. The cleaved caspase-3 primary antibody was detected by a 594-conjugated Alexa Fluor goat anti-rabbit antibody, while the antibody for collagen-IV was detected by 647-conjugated Alexa Fluor goat anti-rabbit antibody (1: 500; Molecular Probes, Eugene, OR, USA). Detection of the NG2-antibody was done by a 594-conjugated Alexa Fluor secondary antibody, the PDGFRβ-antibody was detected by a 594-conjugated Alexa Fluor goat anti-rabbit antibody.

*Vascular leakiness and hypoxia were* evaluated by the formation of pimonidazole adducts *by injection of* FITC-dextran with molecular weight of 2000 kDa (Sigma-Aldrich) into the tail vein of mice prior sacrifice as previously described. For characterization of hypoxia, pimonidazole hydrochloride (Artimmune Analytic GmbH, Kelkheim, Germany) was applied in the presented study as described in detail in a previous publication [16]. The detection was done by the formation of pimonidazole adducts in hypoxic areas of the tumors and their identification with a FITC monoclonal anti-pimonidazole antibody (1: 50; clone 4.3.11.3, Artimmune Analytic GmbH).

### Image acquisition and analysis

The number of vessels examined in 100 × images of tumour sections on 5–10 microscopic images per tumour was quantified as median vessel density (MVD) [15]. Using ImageJ software (U.S. National Institutes of Health, Bathesda, Maryland, USA) size of the vessels was analyzed of 50 vessels per tumour from representative images of a minimum of three different tumours per group. Vessel leakiness was analysed in 100 × images of tumours, where the mice were injected witch FITC-dextran and the number of leaky vessels was characterized. Pericyte coverage was determined by analysing the colocalization of NG2 or PDGFRβ and CD31 as previously described by our group.

### For oxygen diffusion studies

From pimonidazole injected mice a 40 × multi-image alignments (MIA) of tumour sections with a CD31 co-staining were generated. The diameter of each vessel to the rim of the hypoxig region was measured. Oxygenated tissue area per vessel was calculated by measuring the pimonidazole negative area per vessel. Insufficient vessels were hereby defined as vessels that co-localize with pimonidazole positive areas. Using ImageJ, the fraction of hypoxic tumour was calculated as the percentage of area with pimonidazole immunoreactivity per whole tumour section. By measuring the amount of tumour areas that were cl. caspase-3 positive, tumor cell death was evaluated. From at least three tumours per groug, the number of blood vessels with an open lumen was determined from 5 to 10 microscopic images of tumour sections at × 100 magnification. The fraction of open lumen vessels per overall vessel count was analyzed.

### Statistical analysis

All results are expressed as an average ± standard error of the mean (s.e.m.). Between experimental groups diffrences were calculated by unpaired Student’s t-test or one-way ANOVA for growth curves of the tumors. As statistically significant P ≤ 0.05 was considered.

## Results

**Fig. 1:**
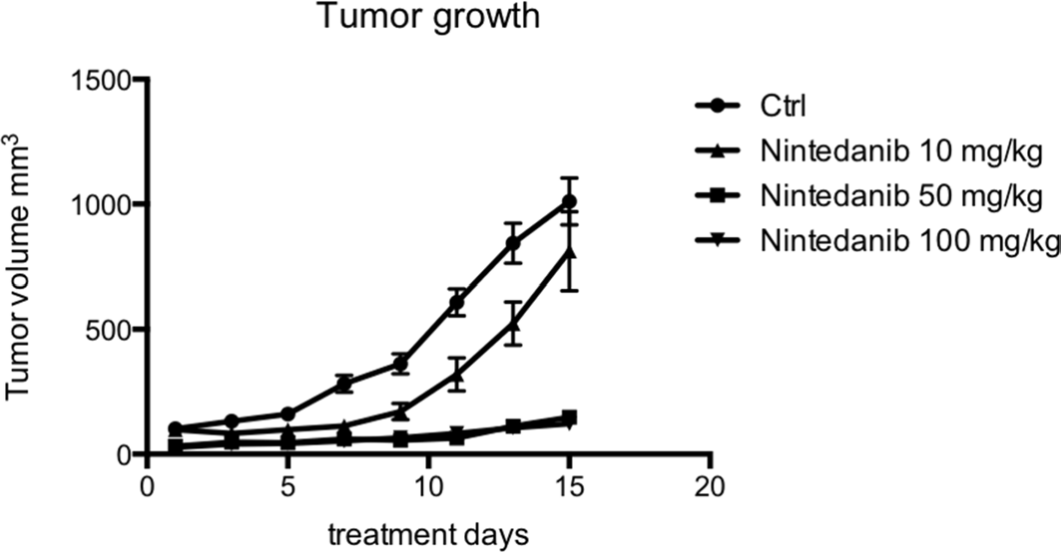
Tumor growth upon Nintedanib treatment in a xenograft model of CRC. Tumor growth curves of subcuteaneous LS174T xenograft tumours. Tumour-bearing mice were treated with vehicle, N10 (10 mg/kg), N50 (50 mg/kg) or N100 (100 mg/kg). To see individual tumor volumes at each time point, see Suppl. Table 1. One-way ANOVA with multple comparisons with Bonferroni's post- hoc test. Ctrl. vs. Nintedanib ns, Ctrl. vs. Ninetanib 50 < 0.05, Ctrl. vs. Nintedanib 100 < 0.05. To see indidivual comparison from day 11 of treatment see Suppl. Table 2

To assess the effects of a combined blockade of VEGFR/PDGFR/FGFR in CRC on tumor growth we employed a xenograft tumour model of VEGF-expressing human colorectal cancer cells (LS174T) under Nintedanib inhibition. The compound was used at three different dosages (N10 (10 mg/kg), N50 (50 mg/kg) and N100 (100 mg/kg) respectively) to evaluate dose response effects. Treatment with nintedanib induced a moderate reduction in tumour growth at the lowest dose N10 compared to the vehicle-treated controls. At the higher Nintedanib doses N50, there was a further significant reduction in tumor growth, but little additional growth reduction was observed at N100. Temporally, the greatest difference in tumour volume between the dose cohorts was seen 14 days after commencing treatment (Fig. 1). This is within the time frame of the suspected impact on tumor angiogenesis.

**Fig. 2:**
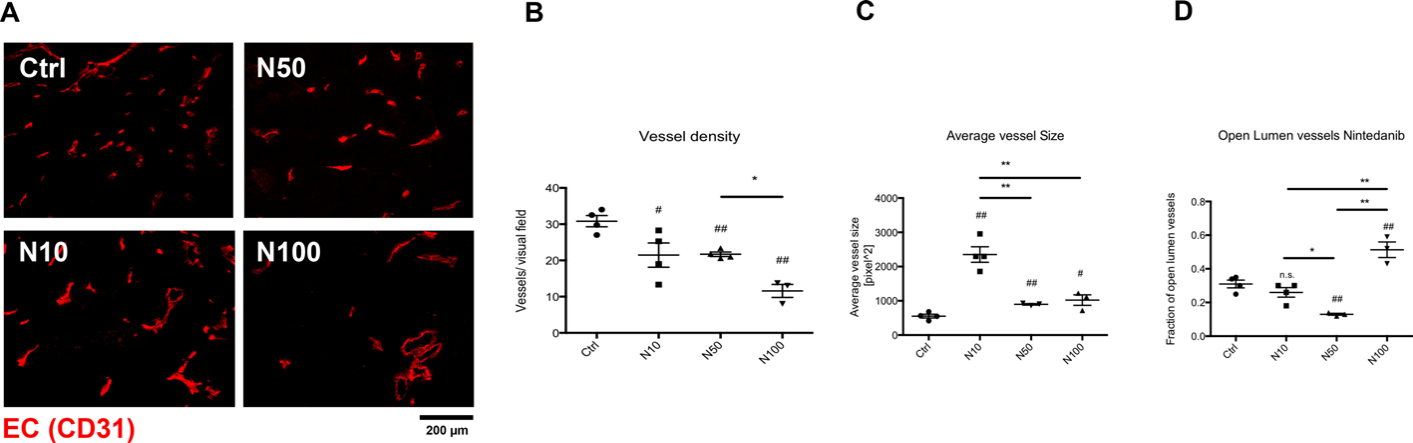
Nintedanib effect on vessel structure. **A** Representative images of CD31 stained cryo sections of subcutaneous CRC tumors treated with different dosages of Nintedanib (Ctrl, N10, N50, N100). **B** Quantification of tumor vessel density. **C** Quantification of average tumour vessel size **D** Measurement of the fraction of intratumoural open lumen vessels. Magnification 100 ×. #, *P < 0.05, ##, **P < 0.01, Asteriks over bar depicts individual group comparisons. rhombus over datapoints depicts comparison to control

**Fig. 3:**
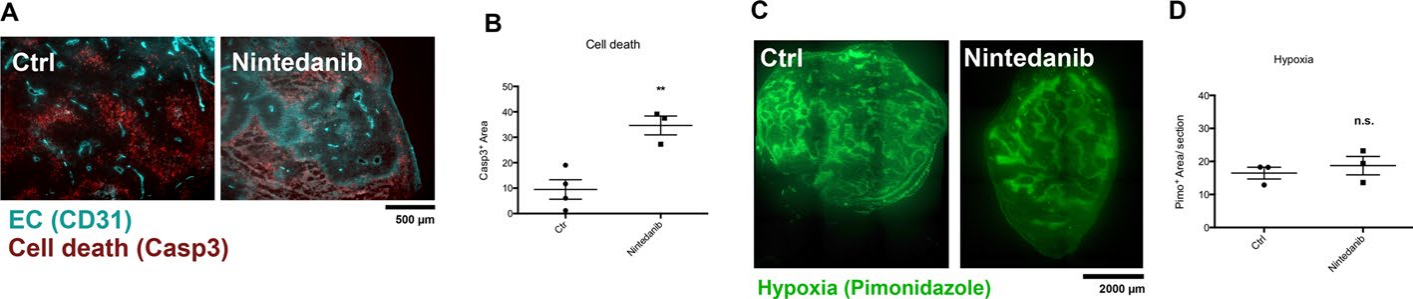
Impact on Hypoxia and cell death upon Nintedanib treatment. **A** Representative images of CD31 and Caspase-3 co-staining of subcutaneous CRC tumors**. B** Quantification of Caspase-3 positive staining per area in Nintedanib treated CRC tumors and controls. **C** Representative images of Pilomidazole staining in CRC tumors treated with Nintedanib and control. **D** Quantification of Pilomidazole positive area per section in CRC tumors treated with Nintedanib and control. Magnification 40x. **P < 0.01, ns: not significant

To verify this hypothesis, tumor vessels were analysed upon Nintedanib treatment using immunofluorescence microscopy on CD31 stained tumor cryo-sections. The tumor vessel density showed a dose dependent reduction upon Nintedanib treatment (Fig. 2A). The average vessel diameter was increased upon Nintedanib treatment but most significantly in the N10 group and to a lesser degree in the higher dose cohorts N50 and N100 (Fig. 2B). We have previously shown that vascular normalization can be associated with an increased fraction of vessels ‘with an open lumen’ [17]. We also analysed this parameter here. A significantly increased fraction of open vessels was only observed in the N100 treated cohort where this fraction reached the majority, but not the N10 and N50 treated cohorts (Fig. 2C, D). To analyze if the observed reduction in tumor vascularity upon Nintedanib treatment impacted cell death, we performed immunfluorescence co-stainings of endothelial cells with CD31 with either areas of cell death with Caspase-3 or hypoxic areas with Pimonidazole (Fig. 2A, C). Nintendanib treatment significantly increased tumor cell death (Fig. 3A, B) while there was no measurable difference between control and nintedanib treated tumors regarding pimonidazole positive hypoxic areas (Fig. 3C, D).

**Fig. 4:**
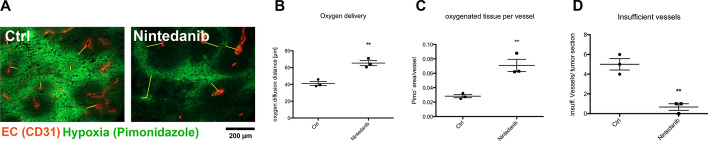
Oxygen delivery upon Nintedanib treatment. **A** Representative images of Pilomidazole and CD31 co-staining of subcutaneous CRC tumors. **B** Measurement of the oxygen diffusion distance in CRC tumors of Nintedanib tumors and controls. **C** Quantification of Pilomidazole positive area per vessel in CRC tumors treated with Nintedanib and control. **D** Quantification of insufficient vessels per tumor section. Magnification 100 ×. **P < 0.01

A parameter of vascular normalization can be increased delivery of oxygen to tumor tissues. Nintedanib treated tumor vessels showed a higher oxygen diffusion distance than controls, meaning that the distance measured from from the vessel wall to the edge of the pimonidazole positive hypoxic area was greater in the Nintedanib group than in vehicle treated controls (Fig. 4A, B). In keeping with this, the area of oxygenated (pimonidazole negative) tissue per vessel count was increased upon Nintedanib treatment compared to controls (Fig. 4C). In line with this, the number of insufficient (poorly perfused) vessels that reside in hypoxic areas and are not surrounded by oxygenated (pimonidazole negative) tissue are increased in the control group compared to Nintedanib treated tumors (Fig. 4D).

**Fig. 5:**
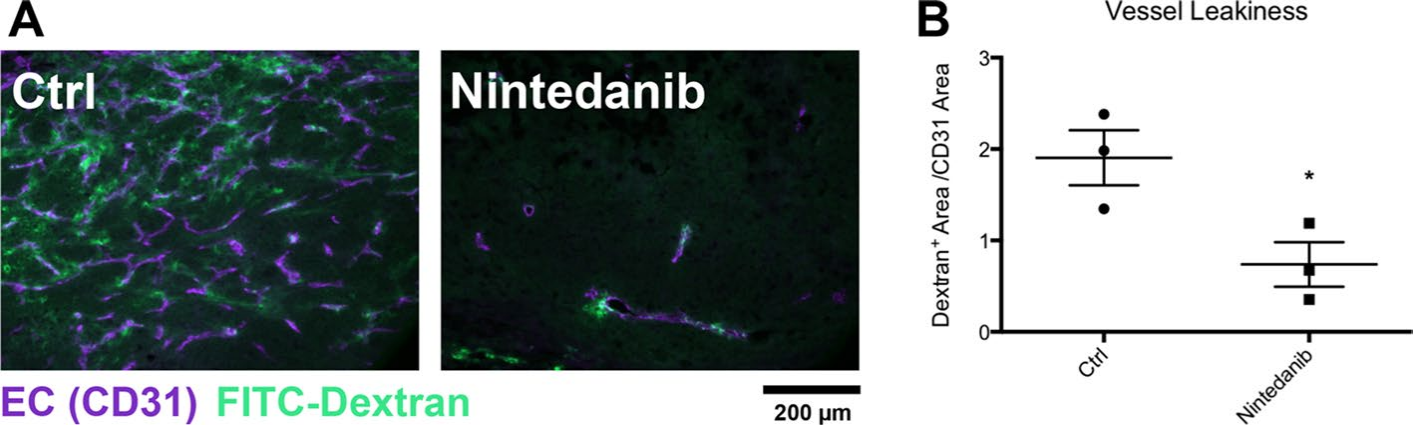
Nintedanib effect on vessel permeability. **A** Representative images of FITC Dextran and CD31 co-staining. **B** Quantification of Dextran positive area per CD31 positive area as surrogate parameter of vessel leakiness in CRC tumors treated with Nintedanib or control. Magnification 600 ×. *P < 0.05

To complement our analyses of normalization parameters we analysed vascular permeability. Tumor bearing animals were intravenousely injected with FITC Dextran before being sacrificed and sections were counterstained with CD31. In the Nintedanib treated tumors we observed a significant reduction in vascular leakage compared with controls, indicaint improved vessel integrity and function as a further surrogate marker for vascular normalization (Fig. 5A and B).

**Fig. 6:**
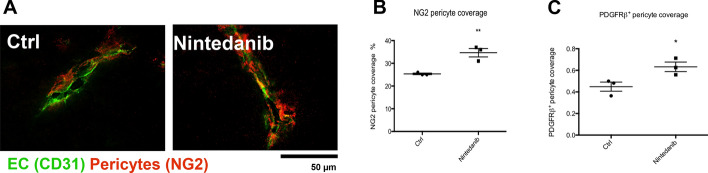
Nintedanib effect on pericyte coverage. **A** Representative images of NG2 pericyte and CD31 co-staining. **B** Quantification of NG2 pericyte coverage. **C** Quantification of PDGFβ pericyte coverage. Magnification 600 ×. *P < 0.05. **P < 0.01

To further evaluate vessel maturation as parameter of vascular normaliszation we analysed pericyte coverage by staining NG2 and PDGFRβ. We found a significant increased pericyte coverage for both markers upon Nintedanib treatment, indicating that vessels that remain after VEGF/PDGF/FGF blockade are significantly more mature than in the control groups (Fig. 6A–C).

## Discussion

In line with previous results obtained with combined inhibition of VEGFR/PDGFR/FGFR either with BIBF 1120 (Nintedanib), our results confirm that Nintedanib inhibits the growth of LS174T xenografts and blocks tumor angiogenesis. Furthermore, we show that treatment with Nintedanib improves oxygen delivery to tumor tissues by the remaining vessels and that Nintedanib reduces leakage of tumor blood vessels. The latter is a surrogate for reduced intratumoural interstital fluid pressure which is critical for drug delivery from vessels to tumor tissue. Both observations suggest that Nintedanib leads to vascular normalization which can improve response to and effectiveness of radio-/chemotherapy for colorectal cancer.

Nintedanib has already been tested preclinically where higher dosages of Nintedanib showed no measurable systemic effect possibly due to the fact that the serum levels were low after 24 h [17].

For metastatic CRC Nintedanib was already applied in clinical trials, e.g. in 14 of 21 patients. These patients showed a reduced vascularity in dynamic contrast-enhanced magnetic resonance imaging upon Nintedanib treatment. The reduction was highly associated with a non- progressive disease [8]. Nonetheless, in a following phase III trial comparing Nintedanib to placebo no benefit was shown on progression-free survival. Importantly, in the Nintedanib group 95.3% and 96.9% of patients in the control group had previously received an anti-angiogenic therapy with bevacizumab, thus, potential Nintedanib associated effects and benefits should be interpreted with caution [18].

Furthermore, in a first- line setting where the chemotherapeutical regimen mFOLFOX6 was combined either with Nintedanib or bevacizumab, patients showed similar efficacy compared with established bevacizumab therapy [19]. Additionally, in the phase III study LUME-Colon I comprised of patients with metastatic or locally advanced inoperable CRC adenocarcinoma Nintedanib treatment showed a small but significant increase in progession free surival in comparison to placebo, while no effect in overall survival was observed [20]. Our work points on a potential clinically relevant mechanism how Nintedanib improves vascular structural quality resulting in improved oxygen and hypothetically improved drug delivery. The LUME-Colon I study did not involve radiotherapy. It would be interesting to see wheter Nintedanib is able to improve response to radiotherapy as the letter depends on oxygenated tissues to work sufficently.

We have previously demonstrated that dosing of anti-VEGF treatment and the relation between anti-VEGF and e.g. anti-PDGF treatment is critical for vascular normalization [16]. Indeed, optimal dosing of anti-angiogenic drugs to maintain the therapeutic window has been a matter of debate from the very beginning of its clinical application [13]. Applied dosages varied between the above-mentioned clinical studies regarding nintedanib between 50 mg once daily to 250 mg twice daily [8, 13, 18], making it unfeasible to draw reliable conclusions for dose dependend effects. Lately, in clinical studies with Nintedanib treatment in idiopathic pulmonary fibrosis or systemic sclerosis the dosage of 150 mg twice daily was preferred [21, 22]. Still, further work has to be done to clarify the correct dosing of Nintedanib in the clinical setting regarding the application in solid tumors.

## Conclusion

Nintedanib showed anti-tumoral and anti-angiogenic effects and lead to vascular normalization in a xenograft mouse model of colorectal cancer. Our findings warrant further investigation of Nintedanib in clinical trials. Nintedanib might be a valuable anti-angiogenic option for patients with refractoriness against ‘conventional’ anti-angiogenic treatments.

## Supplementary Information

Supplementary file1 (XLSX 12 KB)

Supplementary file2 (XLSX 9 KB)

## Data Availability

Datasets generated and analyzed for this work are not publicly available, but are accessible upon request.
